# Case Report: Management of a delayed type III hypersensitivity reaction with acute kidney injury in a dog after administration of human serum albumin with immunoadsorption

**DOI:** 10.3389/fvets.2025.1671034

**Published:** 2025-10-13

**Authors:** Florian Sänger, Fabian Nagel, Saskia Herges, Karin Weber, René Dörfelt

**Affiliations:** LMU Small Animal Clinic, Centre for Clinical Veterinary Medicine, Faculty of Veterinary Medicine, Ludwig-Maximilians-Universität München, Munich, Germany

**Keywords:** immunoglobulin G, antigen–antibody complexes, hypoalbuminemia, intermittent hemodialysis, extracorporeal blood purification, immunocomplexes, serum sickness

## Abstract

A six-year-old, intact male German Shepherd, weighing 43 kg, was presented with generalized edema formation and acute kidney injury due to a suspected delayed type III hypersensitivity reaction and vasculitis 2 weeks after the administration of human serum albumin (HSA). At presentation, the patient had a moderately reduced general condition, a heart rate of 96/min, rectal temperature of 37.0 °C, generalized edema, hematoma and petechiae on all limbs and the abdomen, and scleral hemorrhage. The initial blood work showed a moderate anemia, a severe azotemia, and a moderate hypoalbuminemia. Marked proteinuria with a urine protein-creatinine ratio (UPC) of 6.26 was present. The presence of anti-HSA antibodies was proven with an in-house ELISA. For treatment, immunoadsorption (IA) was performed with the TheraSorb® Ig Omni 1 adsorber, which was integrated in the LIFE 21® apheresis unit. Due to severe azotemia, an intermittent hemodialysis treatment with the dialysis platform Fresenius 4008 was performed after IA. Both treatments were repeated on the following day. A total plasma volume of 1.9 liters and 3.7 liters, respectively, was processed with IA. On the following days, creatinine concentration declined and the patient improved significantly. The patient was discharged after 10 days. UPC decreased to 0.82 and edema completely resolved. Two weeks after discharge, Creatinine, UPC and albumin were in the reference range. IA might be an additional therapeutic option for dogs with severe acute kidney injury due to a suspected delayed type III hypersensitivity reaction.

## Introduction

Albumin is one of the most important proteins in the body. It is responsible for maintaining colloid osmotic pressure, carrying endogenous and exogenous substances, mediating coagulation, and control of oxidative damage (1). Hypoalbuminemia is a common condition in critically ill animals, frequently observed in patients with sepsis, systemic inflammatory response syndrome, protein-losing enteropathy and nephropathy as well as liver failure. In critically ill patients, albumin synthesis is decreased due to a preferential synthesis of acute phase proteins. Furthermore, food intake is typically reduced resulting in inadequate intake of amino acids which are required for albumin synthesis (1, 2). Administration of human serum albumin (HSA) solutions is an established treatment for hypoalbuminemia in dogs. It rapidly increases albumin concentration, but is known to cause anti-HSA antibodies and hypersensitivity reactions (2–5). Antibody generation can be seen in critically ill patients as well as in healthy dogs and can cause immediate or delayed adverse reactions (3). Delayed type III hypersensitivity reactions due to HSA treatment causing lethargy, lameness, edema, petechiae and cutaneous lesions indicative of vasculitis, vomiting, and inappetence are described about 2 weeks after the HSA application (6–8). In some cases, development of acute kidney injury (AKI) is also described (7). In one case series, the deposition of antigen–antibody complexes to HSA in the dermis was demonstrated with immunohistochemical staining (8).

Treatment for the delayed type III hypersensitivity reaction associated with HSA typically includes administration of diphenhydramine, glucocorticoids, and symptomatic treatment (6–8). A case report of a dog with delayed type III hypersensitivity reaction to HSA describes the successful application of therapeutic plasma exchange (TPE) as an alternative treatment option. The dog had signs of vasculitis and AKI and was unresponsive to traditional management with glucocorticoids and fresh frozen plasma (FFP) transfusion. After 3 TPE treatments with a total of 2.7 plasma volumes exchanged, there was a gradual clinical improvement and albumin and creatinine concentrations also improved (9).

Immunoadsorption (IA) is an extracorporeal blood purification technique that is used to eliminate antibodies and circulating antigen–antibody complexes from the blood. The platforms use adsorbers with a matrix-bound ligand that can specifically bind different subclasses of immunoglobulins and circulating antigen–antibody complexes. The blood is separated into plasma and cells and the plasma is provided through the adsorber. IA has been described in a few case reports in dogs with immune-mediated diseases caused by auto-antibodies and is known to reduce circulating antibodies (10–12). The present case report describes the successful application of IA in a dog with a delayed type III hypersensitivity reaction, with coagulopathy and AKI, 2 weeks after HSA treatment.

## Case presentation

A six-year-old, male, intact, German Shepherd, weighing 43 kg, was presented due to generalized edema formation and AKI. One month ago, the dog was hospitalized in another clinic due to an intestinal volvulus. The volvulus could be managed surgically and the dog was discharged 3 days later. Two days after discharge, the dog was presented again with anorexia, fever, and bloody diarrhea, due to an ileocolic intussusception. After surgery, the dog developed hypoalbuminemia with generalized edema and was treated with transfusion of whole blood and a HSA solution of an unknown dose.

**Figure 1:**
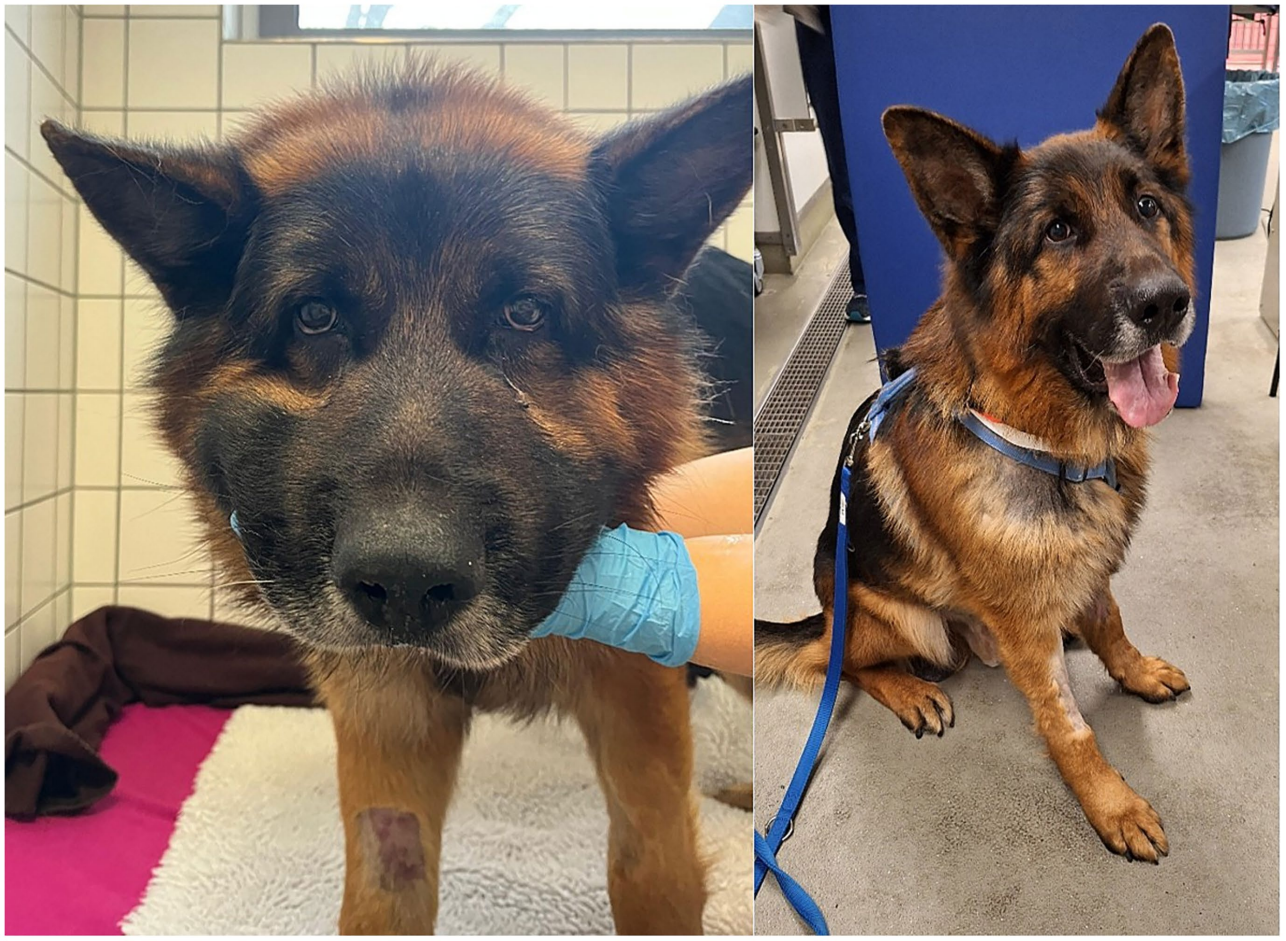
Clinical picture of the patient on the day of admission (left side) with generalized edema and on the day of discharge (right side) with restored normal appearance.

Two weeks after the HSA treatment, the dog developed generalized edema and AKI due to a suspected delayed type III hypersensitivity reaction and vasculitis because of the HSA treatment. The dog was referred for extracorporeal blood purification therapy. During the initial physical examination, the patient had a moderately reduced general condition, a heart rate of 96/min, pink mucous membranes with a capillary refill time of < 2 s, rectal temperature of 37.0 °C and generalized edema. As the normal body weight of the dog was about 39 kg, a fluid overload of 4 liters was estimated. The patient showed multiple hematomas and petechiae on all limbs and the abdomen, and scleral hemorrhages (Figure 1). The initial blood gas analysis showed a moderate metabolic acidosis (pH 7.24; reference range: 7.3–7.45; bicarbonate 13.9 mmoL/L; reference range: 18–25 mmoL/L) with respiratory compensation (pCO_2_ 30 mmHg; reference range: 32–51 mmHg), a mild hyponatremia (136 mmoL/L; reference range: 140–152 mmoL/L) and hypochloremia (103 mmoL/L, reference range: 106–119 mmoL/L) and a mild hyperkalemia (5.5 mmoL/L, reference range: 3.3–4.7 mmoL/L). Hematology (CBC) showed a moderate non-regenerative anemia (hematocrit 23.1%, reference range: 37–61%; reticulocytes 26.3 K/μl, reference range: 10–110 K/μl; MCV 67.7 fl, reference range: 62–74 fl; MCHC 34.6 g/dL, reference range: 32–38 g/dL). Biochemistry profile showed a severe azotemia with increased creatinine (612 μmoL/L, reference range: 44–125 μmoL/L) and urea (64.1 mmoL/L, reference range: 3.5–10.8 mmoL/L) and hypoalbuminemia (21.4 g/L, reference range: 31.3–43.0 g/L). In the urinalysis, marked proteinuria with a urine protein-creatinine ratio (UPC) of 6.26 was present. Urine specific gravity was 1,013 and urine sediment was unremarkable.

Initial treatment consisted of intravenous fluid therapy with an isotonic crystalloid at a rate of 2 mL/kg/h, maropitant (1 mg/kg IV q 24 h; Prevomax, Dechra Pharmaceuticals, Northwich, United Kingdom), metoclopramide as a constant rate infusion of 60 μg/kg/h IV (Emeprid; Ceva, Libourne, France), chlorphenamine (0.5 mg/kg IV q 12 h; Phenasol, CP-Pharma, Burgdorf, Germany), buprenorphine (20 μg/kg IV q 6 h; Bupresol, CP-Pharma, Burgdorf, Germany) and prednisolone (1 mg/kg PO q 24 h; Prednitab 20 mg, CP-Pharma, Burgdorf, Germany).

Due to the high suspicion of a delayed type III hypersensitivity reaction on HSA, IA to reduce antigen–antibody complexes in this patient, followed by intermittent hemodialysis (IHD), was initiated. For this treatment, a 14 French, 25 cm double-lumen central venous catheter (Arrow Germany GmbH, Erding, Germany) was inserted in the right jugular vein and correct position was confirmed with radiography after that. IA was performed with the TheraSorb® Ig Omni 1 adsorber (Miltenyi Biotec B.V. & Co. KG, Bergisch Gladbach, Germany), which was integrated in the LIFE 21® apheresis unit (Miltenyi Biotec B.V. & Co. KG, Bergisch Gladbach, Germany). This platform works with two adsorbers that are used alternately. The plasma is separated by centrifugation during each cycle, then passed through the adsorber, and finally recombined with the cells before it is returned to the patient. During load of one adsorber, the other adsorber is regenerated for the next cycle. Extracorporeal blood volume was 160 mL. Tubing was used and primed according to the manufacturer’s instructions with isotonic saline. Anticoagulation was performed with an initial bolus of heparin of 20 IU/kg IV (Heparin-Natrium 25,000 I. E./5 mL Inj. Lsg., B. Braun Vet Care AG, Melsungen, Germany) and citrate (ACD-A, Fresenius Kabi, Bad Homburg, Germany) at an initial blood/citrate ratio of 1/20 in the beginning, which was reduced to 1/24 after 30 min. To avoid hypocalcemia, the dog received calcium gluconate 10% (Calciumgluconate 10%, B. Braun Melsungen GmbH, Melsungen, Germany) via a peripheral venous catheter, according to a protocol established for hemodialysis. Calcium concentration in the circuit and in the patient were checked after 30 min, 60 min and then every hour. Machine settings and blood flow of 50 mL/min were selected to process 3.7 L of plasma in 60 immunoadsorption cycles with 50 mL each (1.5-fold plasma volume). Due to clotting in the tubing system and the adsorbers, the first IA was prematurely stopped after 1.9 L of plasma were processed within 33 cycles. A positive fluid balance of 1,100 mL was achieved after treatment.

IHD was performed to decrease severe fluid overload and to reduce uremic signs. For IHD, the dialysis platform Fresenius 4008 (Fresenius Medical Care GmbH und Co KG, Bad Homburg, Germany) with the dialyzer Fresenius FX 60 classix (Fresenius Medical Care GmbH und Co KG, Bad Homburg, Germany) was used. The IHD treatment prescription was as followed: commercial dialysate acid concentrate with K 2.0 mmoL/L and Ca 0 mmoL/L, dialysate flow 500 mL/min, blood flow rate between 80–140 mL/min, and a target urea reduction ratio (URR) of 50%. During the treatment, a total blood volume of 19.6 L was processed during 2.5 h. Anticoagulation was performed with heparin as a bolus of 30 IU/kg IV followed by regional citrate anticoagulation with calcium support as described for the IA treatment. A total ultrafiltration volume of 2.5 L was achieved in this session. The dog was monitored during both procedures with electrocardiography and non-invasive blood pressure with a multi-parameter monitor (IntelliVue MP50, Philips). All vital parameters were within the physiological range during the treatments.

Blood gas analysis, CBC and a biochemistry profile were performed on the next day and results were similar to the day before. Due to the suspected low efficacy of the first treatment, a second treatment with IA and IHD was performed on this day. Prescription for IA was the same as for the first treatment. During the second IA treatment, the 3.7 L of plasma were processed within 60 immunoadsorption cycles with 50 mL each (1.5-fold plasma volume). Additional anticoagulation with heparin boluses of 40 IU/kg every hour IV was performed. A positive fluid balance of 1,600 mL was achieved after treatment.

For the second IHD treatment, the dialyzer Fresenius FX 80 classix (Fresenius Medical Care GmbH und Co KG, Bad Homburg, Germany) was used. The treatment prescription was as followed: commercial dialysate acid concentrate with K 1.0 mmoL/L and Ca 1.25 mmoL/L, dialysate flow 500 mL/min, blood flow rate between 80 and 140 mL/min, and a target urea reduction ratio (URR) of 60%. During the second IHD treatment, a total blood volume of 28.1 L was processed within 3.5 h. A total ultrafiltration volume of 3.5 L was achieved in this session. Anticoagulation was performed with a heparin constant rate infusion of 40–70 IU/kg/h IV. Anticoagulation was monitored with aPTT measurement after 30 min and then every hour.

After IA, the dog was further treated with maropitant, metoclopramide, chlorphenamine, buprenorphine and prednisolone. In addition, the dog received esomeprazole (1 mg/kg IV q 12 h; Nexium; Grünenthal, Aachen, Germany), ondansetron (0.2 mg/kg IV q 8 h; Ondansetron-hameln, Hameln Pharma Plus, Hameln, Germany) and amlodipine (0.2 mg/kg PO q 12 h; Amodip 1,25 mg, Ceva, Brussel, Belgium) due to systemic hypertension with a systolic blood pressure of about 200 mmHg.

**Figure 2:**
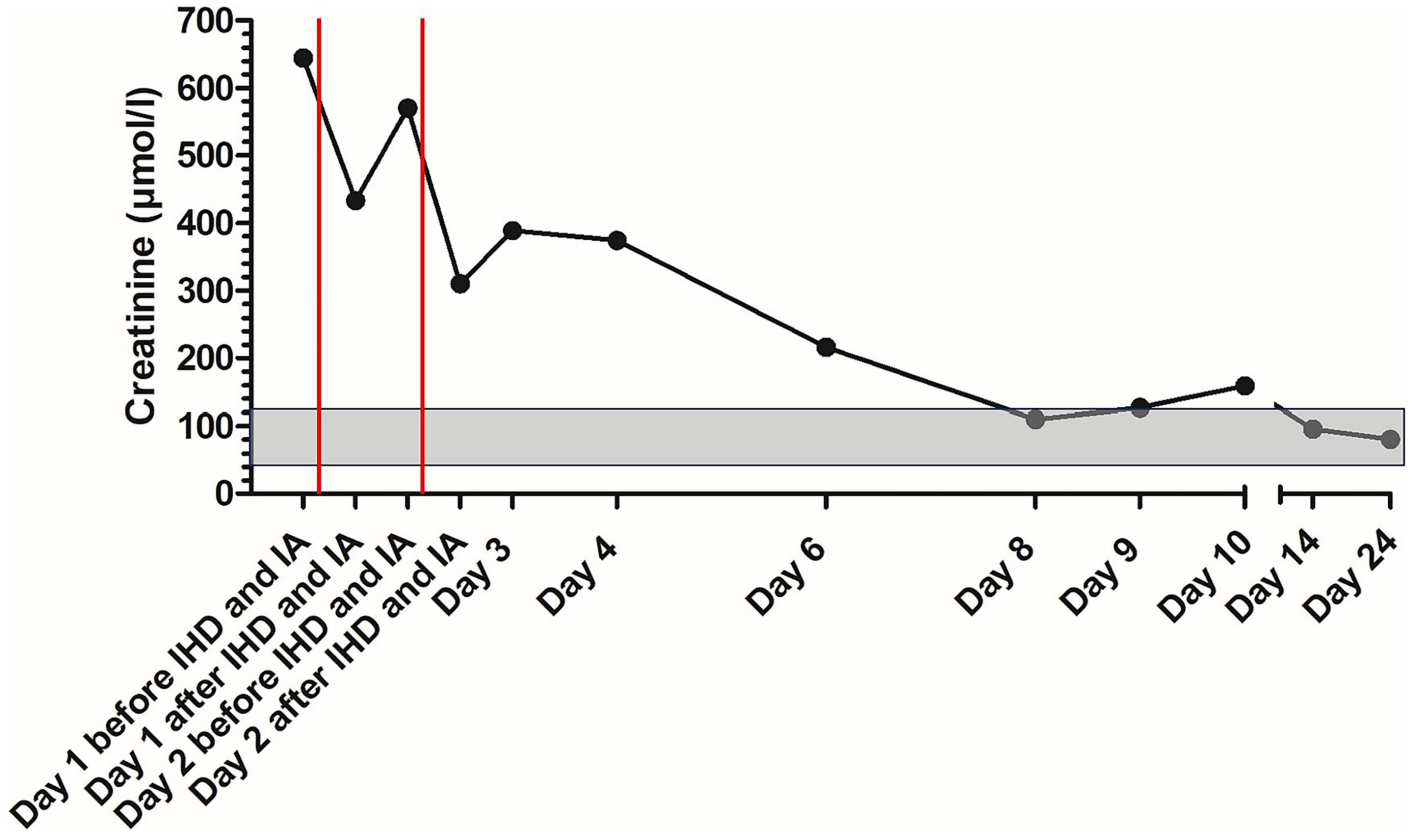
Course of creatinine concentration in the patient with delayed type III hypersensitivity reaction to human serum albumin during treatment; the gray area marks the reference range; the red lines mark the time points of immunoadsorption. IA, immunoadsorption; IHD, intermittent hemodialysis.

**Figure 3:**
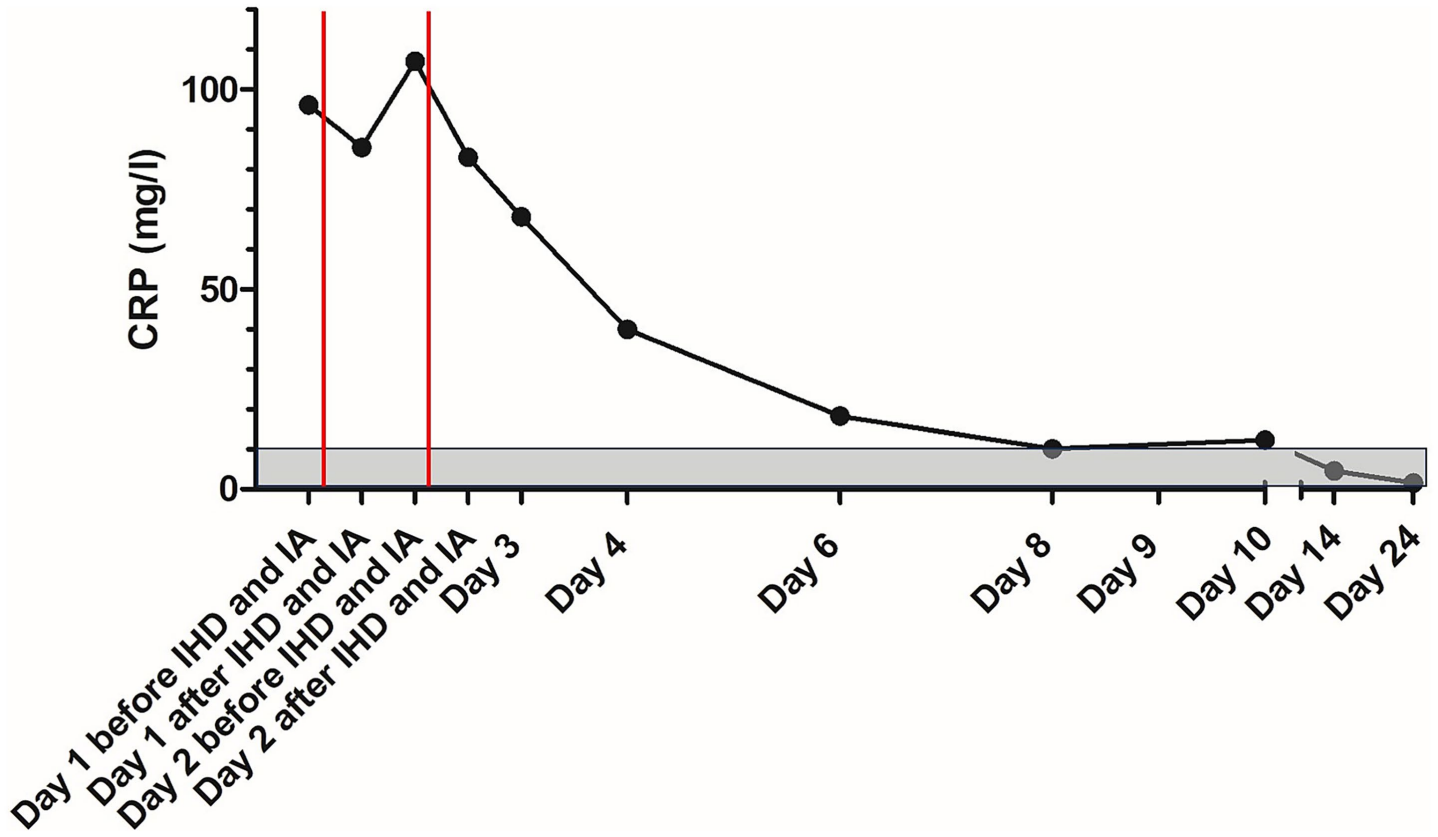
Course of C-reactive protein concentration in the patient with delayed type III hypersensitivity reaction to human serum albumin during treatment; the gray area marks the reference range; the red lines mark the time points of immunoadsorption. CRP, C-reactive protein; IA, immunoadsorption; IHD, intermittent hemodialysis.

On the following days, creatinine concentration declined and the patient improved significantly (Figure 2). Initially elevated C-reactive protein (CRP) concentration increased slightly after IA and IHD and declined on the following days (Figure 3). The patient was discharged after 10 days. UPC on the day of discharge decreased to 0.82 and edema completely resolved (Figure 1). The dog was discharged with amlodipine and prednisolone. Prednisolone was tapered over the next 6 weeks. Blood pressure was regularly measured and amlodipine could also be reduced over the next weeks and could finally be discontinued.

For confirmation of a hypersensitivity reaction on HSA, an ELISA was implemented. Nunc Maxisorb ELISA plates and an ELISA buffer kit (ThermoFisher/Invitrogen) with coating buffer, assay buffer, wash buffer, stabilized chromogen and stop solution were used to detect anti-HSA antibodies. Controls with blank wells, wells with HSA only, and wells without HSA-coating but with serum samples from the patient and two control dogs were included. All samples and controls were measured in duplicates. The plates were coated with 50 mM carbonate buffer pH 9.4 (Coating Buffer B) containing 5 μg/mL HSA (Merck) for 6 h at 4 °C. Plates were washed 3 times with wash buffer and blocked overnight at 4 °C with assay buffer. The assay/blocking buffer was removed, and serum samples of the patient and of 12 control dogs that were expected to be negative for anti-HSA antibodies were diluted 1:500 in assay buffer and incubated on the plates for 1 h at room temperature (RT). The plates were washed 3 times and incubated with a peroxidase-conjugated Rabbit Anti-Dog IgG (Merck) diluted 1:2000 in assay buffer for 1 h at RT. The plates were washed 6 times and incubated with the stabilized chromogen (tetramethylbenzidine and hydrogen peroxide) for 3 min. The stop solution (sulfuric acid) was added and absorbance was measured at 450 and 595 nm in a Biotek plate reader. The results are given as the optical density (OD) mean of the duplicates of the absorbance reading difference 450–595 nm. HSA-only wells gave a reading below 0.01 OD, all wells without HSA-coating and all samples from control dogs gave a reading below 0.2 OD, confirming the specificity for anti-HSA antibodies in the patient serum samples (all readings above 1.7 OD). All positive duplicate measurements (OD > 1.7) had a coefficient of variation (CV) below 2.3%, all negative duplicate measurements (OD < 0.17) had a CV below 16%. This intra-assay performance was considered acceptable, since CV > 10% only occurred in the very low OD range, and no repeats were required.

**Figure 4:**
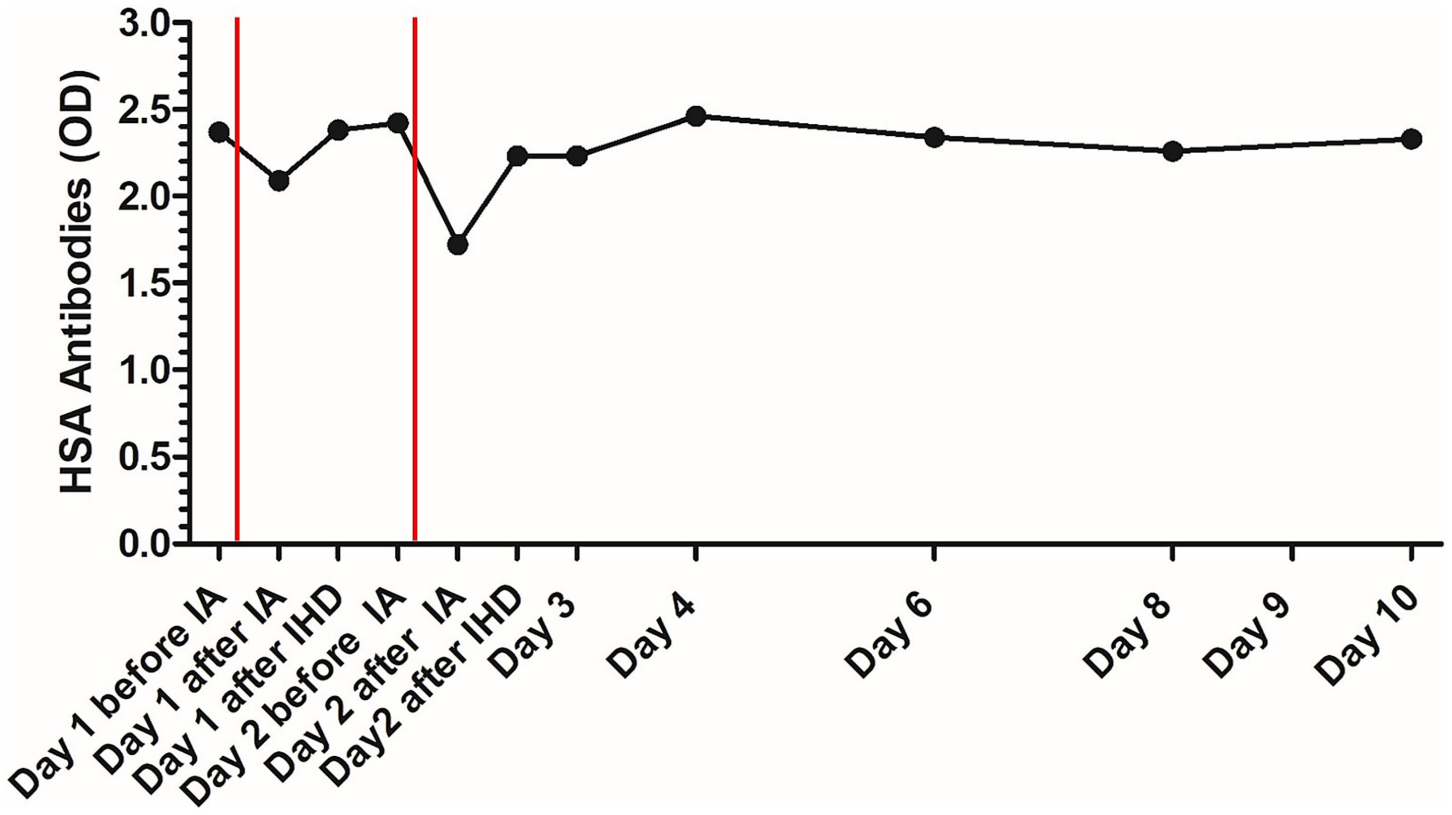
Course of human serum albumin antibody concentration in the patient with delayed type III hypersensitivity reaction to human serum albumin during treatment; the red lines mark the time points of immunoadsorption. HSA, human serum albumin; OD, optical density; IA, immunoadsorption; IHD, intermittent hemodialysis.

Initially elevated anti-HSA antibodies decreased about 12% with the first IA session and 29% with the second IA session, but increased again after IHD and on the following day in both sessions. After the rebound on the day after the second IA treatment, anti-HSA antibody concentrations remained stable until discharge (Figure 4).

Two weeks after discharge, the patient was presented for a check-up at his primary care veterinarian. The dog was in excellent condition and Creatinine, CRP, UPC and albumin were in the reference range.

## Discussion

This is the first report of IA in a dog with a delayed type III hypersensitivity reaction to HSA. In veterinary medicine, four categories of hypersensitivity reactions are described: type I (immediate), type II (antibody-mediated), type III (immunocomplex-mediated), and type IV (cell-mediated). The first three types (type I – III) are immunoglobulin-dependent reactions involving a humoral B-lymphocyte-mediated response, whereas type IV is a T-cell-mediated hypersensitivity reaction (13). In type III hypersensitivity reactions, an increased production of antigen–antibody complexes occurs due to contact with a foreign antigen, and these antigen–antibody complexes are deposited in various tissues (13). This deposition of excess antigen–antibody complexes is known as serum sickness. Typical diseases associated with type III hypersensitivity are immunocomplex-mediated glomerulonephritis, vasculitis and arthritis (13). The development of immunocomplex-mediated glomerulonephritis as well as vasculitis after HSA application is already described in dogs (6, 7, 9). The history, symptoms and high UPC of the dog presented in this case report are consistent with previous case reports and support, in conjunction with the high concentration of anti-HSA antibodies, the diagnosis of a delayed type III hypersensitivity reaction. Nevertheless, a definitive histopathological diagnosis of a glomerulonephritis with a kidney biopsy and immunohistochemical staining has not been performed in this dog.

The effect of IA on the reduction of circulating antigen–antibody complexes is well-known in human medicine (14). IA is successfully used for multiple hematological, neurological, and other systemic immunological disorders. It is effective in patients with systemic immunological diseases affecting the kidney, such as systemic lupus erythematosus, and for the prevention of transplant rejection and graft versus host diseases after blood-type incompatible renal transplantation (15). In veterinary medicine, studies evaluating the effect of IA in different immunological diseases are lacking. There are only a few case reports and a scientific abstract describing the potential benefits of IA in veterinary medicine. The case reports applied IA to dogs with immune-mediated hemolytic anemia, fulminant acquired myasthenia gravis and leishmaniosis-induced glomerulonephritis (10–12). The abstract showed Ig reduction with IA in a few dogs (16). The adsorber Ig Omni 1 used in this case report can bind the immunoglobulin (Ig) subtypes IgG, IgM and IgA as well as circulating antigen–antibody complexes. As delayed type III hypersensitivity reactions to HSA depend mainly on antigen–antibody complexes and their deposition in the tissues, adsorption of these antigen–antibody complexes with IA is likely possible. Therefore, IA is a potential therapeutic approach to reduce circulating antigen–antibody complexes and fatal complications following the deposition of these antigen–antibody complexes in type III hypersensitivity reactions.

The use of extracorporeal blood purification is already described in a case report of a dog with type III hypersensitivity reaction to HSA. A 3-year-old mixed breed dog developed an immune-mediated vasculitis and AKI 2 weeks after the administration of HSA for the management of hypoalbuminemia resulting from septic peritonitis. The dog was treated with 3 TPE sessions over 4 days. A total of 2.7 times the plasma volume was exchanged. Creatinine concentration and clinical signs improved after the TPE treatments. The dog was discharged after 8 days (9). In the present case report, during the first IA treatment, the total plasma volume of the dog was processed once, during the second IA treatment, 1.5 times the plasma volume of the patient was processed. Following major improvement in clinical signs, creatinine concentration and CRP concentration, the dog was discharged after 10 days of hospitalization. Treatment success is comparable to the case report using TPE treatment, but it has to be considered that the dog in the previous case report had a lower creatinine concentration at presentation compared to the dog presented here. For TPE usually large amounts of donor plasma are needed, which are not easily available in all regions, and is therefore a limited resource. As for IA, no donor plasma is required, it could be a more easily available alternative to TPE.

Initially elevated anti-HSA antibodies decreased with the two IA sessions, but increased again after IHD and on the following day. After the rebound on the day after the second IA treatment, anti-HSA antibody concentrations remained stable until discharge. Therefore, anti-HSA antibody concentrations are a parameter for the diagnosis of a hypersensitivity reaction due to HSA treatment, but they do not seem to be suitable for monitoring of the disease process. The drop in anti-HSA antibody concentrations after IA also shows the effect of IA in reducing anti-HSA antibodies. The effect did not last for a long time, probably due to redistribution from the interstitial into the vascular compartment. This increase in antibody concentration on the day after treatment could also be observed in the case report about the dog with myasthenia gravis treated with immunoadsorption. Acetylcholin-receptor antibody concentration was reduced with IA, but increased on the days after IA (11).

The volume of ultrafiltration for both sessions was high in this patient. There were two reasons for that decision. First, the IA platform used in this case is not able to provide any ultrafiltration. Additionally, it always provides a positive fluid balance due to rinse back solution and the volume of anticoagulation during the treatment. A positive fluid balance of 1,100 mL and 1,600 mL, respectively, was achieved in these sessions. This positive fluid balance was planned to be removed immediately with the IHD session. Second, the patient was already severely overhydrated on arrival which should be corrected with ultrafiltration. Therefore, uncommonly high ultrafiltration rates were chosen in this patient. No adverse effects were seen with the high ultrafiltration rate achieved in this patient.

Systemic heparinization is usually not recommended in patients with coagulopathy. As this patient had clinical signs of bleeding like hematomas and petechiae, a coagulopathy might be suspected. During the first IA treatment, calcium concentration in the patient and in the circuit were monitored and were within recommended limits. Despite adequate citrate anticoagulation, clotting in the system occurred during this treatment. Therefore, we decided to add intermittent heparin bolus treatment in addition to the citrate anticoagulation in the second IA session to optimize anticoagulation during treatment.

The influence of IA on the course of disease could not be completely objectively determined. It is possible that the patient’s kidney values would have improved with hemodialysis alone. However, proteinuria, which was severe in this patient, is caused by the deposition of antigen–antibody complexes and consecutive damage of the glomeruli by inflammation. It is unlikely that hemodialysis alone cured glomerulonephritis and proteinuria.

Another limitation is that the ELISA used for the detection of anti-HSA antibodies in this dog is not commercially available, and a positive control standard does not exist. The test was designed based on earlier publications describing HSA antibody detection in dog serum (3, 4). Since all negative controls showed no or very low OD readings, our in-house ELISA appears to be specific for the detection of anti-HSA antibodies.

## Conclusion

This case report shows the potential of IA in patients with severe hypersensitivity reactions. IA might be an opportunity to reduce antibodies and circulating antigen–antibody complexes in dogs with delayed type III hypersensitivity reactions that could not be controlled with traditional management. Further studies are needed to evaluate the effect of IA on delayed type III hypersensitivity reactions in dogs after HSA application.

## Data Availability

The original contributions presented in the study are included in the article/supplementary material, further inquiries can be directed to the corresponding author.
